# Effect of Δ9-tetrahydrocannabinol and cannabidiol on myofascial pain modulation in patients with temporomandibular disorder: a prospective crossover study

**DOI:** 10.1016/j.clinsp.2026.100885

**Published:** 2026-02-24

**Authors:** Francisco Gomes Bonetto Schinko, Luiz Renato Paranhos, Lucas Gonçalves de Sousa, Gabriel Phelipe de Paula Santos, Sigmar de Mello Rode, Antonio Sergio Guimarães, Juliana Cama Ramacciato

**Affiliations:** a Postgraduate Program in Pain and Temporomandibular Disorders, Faculdade São Leopoldo Mandic, Campinas, SP, Brazil; b Department of Orthodontics, School of Dentistry, Universidade Federal de Uberlândia (UFU), Uberlândia, MG, Brazil; c Postgraduate Program in Dentistry, Faculty of Dentistry, Universidade Federal de Uberlândia, Uberlândia, MG, Brazil; d Department of Dental Materials and Prosthesis, Science and Technology Institute, Universidade Estadual Paulista, São José dos Campos, SP, Brazil; e Experimental Pain Laboratory, Faculdade São Leopoldo Mandic, Campinas, SP, Brazil; f Division of Pharmacology, Anesthesiology, and Therapeutics, Faculdade São Leopoldo Mandic, Campinas, SP, Brazil

**Keywords:** Temporomandibular disorders, Cannabidiol, Δ9-tetrahydrocannabinol, Myofascial pain syndromes

## Abstract

**Background:**

Temporomandibular Disorder (TMD) often causes chronic orofacial pain and functional limitations, with conventional treatments providing suboptimal results. Phytocannabinoids such as Δ9-Tetrahydrocannabinol (Δ9-THC) and Cannabidiol (CBD) have analgesic and anti-inflammatory properties, but evidence in TMD is scarce.

**Objective:**

To evaluate the efficacy of Δ9-THC/CBD therapy in reducing pain and improving mandibular function in TMD patients.

**Methods:**

Twenty adults with chronic myofascial pain (DC/TMD diagnosis) participated in a blinded, crossover, non-randomized study. Participants underwent two consecutive 90-day phases: placebo followed by Δ9-THC/CBD therapy (1:1 ratio, starting with a dose of 2 mg/day in the first week, gradually adjusting an increase of 2 mg/week until reaching 10 mg/day in the fifth week, sublingually), without washout. Outcomes included pain intensity (VAS), muscle sensitivity (algometry), mandibular function (mouth opening, protrusion, laterality) and pain sensitivity (allodynia/hyperalgesia). Data were analyzed using linear mixed models for repeated measures.

**Results:**

Δ9-THC/CBD improved all outcomes versus baseline and post-placebo (*p* < 0.001; Cohen’s *d* > 0.8). Mouth opening increased from 45.9 mm to 49.9 mm; VAS pain decreased from 7.35 to 3.50. Functional pain dropped by ∼90%, with near elimination of allodynia and hyperalgesia. Placebo effects were minimal.

**Conclusion:**

Δ9-THC/CBD therapy provided substantial analgesic and functional benefits in TMD patients, supporting its potential as a therapeutic alternative. Larger randomized studies are recommended to validate these findings and explore underlying mechanisms.

## Highlights

• Δ9-THC/CBD reduced TMD pain from 7.35 to 3.50 on the VAS scale.
• Functional gain: mouth opening increased from 45.9 mm to 49.9 mm.
• Significant improvement in mandibular protrusion and laterality.
• ∼90 % reduction in functional pain; allodynia and hyperalgesia nearly absent.
• Superior analgesic effect vs. placebo, with large effect size (*d* > 0.8).

## Introduction

Temporomandibular Disorder (TMD) is a set of conditions that affect the temporomandibular joint, masticatory muscles, and associated structures. It manifests as orofacial pain, functional limitation, and impaired quality of life in patients.^1^ The National Institute of Dental and Craniofacial Research (2025)^2^ reports that TMD affects a significant portion of the world population, with an estimated prevalence of 5 % to 12 %, representing a relevant public health problem. The clinical management of TMD is challenging, given its multifactorial features and the diversity of clinical manifestations.^1^

In recent years, alternative therapies have been explored for treating chronic pain associated with TMD, especially considering the limitations of conventional pharmacological approaches, which often have adverse effects and variable efficacy.^3^ In this context, cannabinoids have emerged as potential therapeutic agents, with increasing evidence indicating their properties as analgesics, anti-inflammatories, and central nervous system modulators.^4^ Clinical studies and systematic reviews have demonstrated the efficacy of cannabinoids in several chronic painful conditions, including musculoskeletal pain, supporting their viability as an alternative for pain management.^5^^,^^6^

A previous systematic review^7^ investigated published evidence on the effects of cannabinoids (natural and synthetic) in the management of postoperative and/or out-of-office pain in patients suffering from orofacial pain. Further studies were recommended by the researchers, as of the five articles included, one reported a significant effect on pain relief from temporomandibular dysfunction using a topical cannabidiol formulation compared to a placebo. Four articles reported no significant effects of cannabinoids on pain management in various orofacial pain conditions. Although one study reported a positive effect, there is insufficient evidence to support a tangible clinical benefit of cannabinoids in the treatment of orofacial pain. However, there are still knowledge gaps concerning specific cannabinoid applications in TMD patients, especially regarding the assessment of their effects on mandibular functional improvement and the reduction of pain symptoms typical of this condition. Thus, it is essential to investigate the therapeutic potential of these compounds in this clinical context, expanding the available treatment options. Therefore, this study evaluated the efficacy of cannabinoid therapy in reducing pain and improving mandibular function in TMD patients, helping to establish clinical evidence to support its safe and effective use for managing this pathology.

## Materials and methods

### Study setting and design

O presente estudo foi delineado e relatado de acordo com as recomendações das diretrizes CONSORT (Consolidated Standards of Reporting Trials) ^8^ de modo a assegurar a padronização metodológica, a transparência na comunicação dos achados e a qualidade do relato científico.

This study was conducted at the postgraduate Temporomandibular Disorders Clinic of São Leopoldo Mandic College, in Campinas, São Paulo, Brazil.

This was a blinded, non-randomized, crossover exploratory clinical trial with a feasibility and preliminary efficacy framework, consisting of two consecutive 90-day intervention phases (placebo and phytocannabinoid). The absence of a washout period was justified by the long duration of each phase and the expected stabilization of pharmacological effects.

No changes were made to the study design or outcome measures after study commencement.

### Ethical aspects

The Human Research Ethics Committee of São Leopoldo Mandic College (SLMADIC) approved the research under opinion n° 6.317.731 (CAAE: 71,674,023.8.0000.5374). This document was prepared in accordance with the ethical principles established by the Declaration of Helsinki and the guidelines of the Brazilian National Health Council (Resolution n° 466/2012).

All participants included in this study provided free and informed consent to take part in the research after receiving detailed information regarding its objectives, procedures, potential risks, and benefits. Consent was obtained individually and in person through the physical signing of the Informed Consent Form. Accordingly, all patients authorized the use of their information for scientific purposes only, ensuring the anonymity and confidentiality of the collected data.

Patients were not involved in the design, conduct, reporting, or dissemination of this study.

### Eligibility criteria

Inclusion criteria required a diagnosis of chronic myofascial pain according to the Diagnostic Criteria for Temporomandibular Disorders (DC/TMD) .^9^ Individuals without this diagnosis, pregnant and breastfeeding women, psychoactive substance users, and those with a history of hypersensitivity or allergy to any component of the pharmacological intervention were excluded.

The study included 20-adult patients of both sexes (≥ 18-years), with an average age of 44.47 years, recruited from the postgraduate clinic of temporomandibular disorders at SLMADIC, in Campinas, São Paulo, Brazil. The screening process was systematic, using the Random.org tool and based on the scheduling list of assisted patients.

### Sample size

The sample size was based on methodological guidelines for pilot studies in clinical trials,^10^^,^^11^ as well as previous studies on TMD with a similar number of participants.^12^ The number of 20 participants was adequate to generate preliminary effect estimates and evaluate the feasibility of the design.

### Interventions

**Fig. 1:**
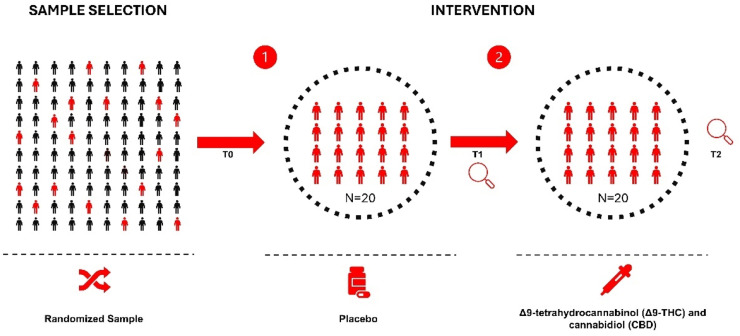
Flowchart representing the design and composition of the clinical study sample; baseline (T0), after 90-days of placebo use (T1) and after 90-days of cannabinoid use (T2).

After the baseline assessment, participants used the placebo (Drug Control Treatment - DCT) for 90 consecutive days, after which they transitioned directly to the active intervention phase with phytocannabinoids for another 90-days, totaling 180-days of follow-up (Fig. 1).

The methodological decision not to include a washout period between phases was based on the long duration of each phase, the continuous monitoring of participants, and the expected stabilization of intervention effects, considering the pharmacological profile of the compounds. The pharmacological intervention consisted of an extract with a 1:1 ratio of Δ9-Tetrahydrocannabinol (Δ9-THC) and Cannabidiol (CBD), administered sublingually at 10 mg/day, divided into two daily doses of 5 mg (15-drops). The treatment started with a dose of 2 mg/day during the first week, gradually increasing by 2 mg/week until reaching 10 mg/day in the fifth week.

During the study, the participants were followed up through text messages every 20 days and reassessed in person at the end of each phase (at 90- and 180-days).

### Clinical outcomes

The outcomes were assessed at three time points: T0 (baseline), T1 (after 90-days of placebo use), and T2 (after 90-days of cannabinoid use). The primary outcomes included: 1) Pain intensity, measured using a Visual Analogue Scale (VAS) ranging from 0 to 10; 2) Painful sensitivity (allodynia/hyperalgesia), identified through an evaluation form completed by the examiner, which recorded clinical examination data (palpation) and responses to direct questions regarding the presence or absence of the condition; and 3) Presence of referred pain, assessed through calibrated digital palpation using a digital algometer (MedEOR® Medtech Ltda) at myofascial trigger points in the masseter muscle and in the anterior and middle portions of the temporalis muscle.

The secondary outcomes concerned mandibular functions and included: 1) Maximum mouth opening, measured with a millimeter ruler and a digital caliper between the incisal edges of the upper and lower central incisors, excluding the overbite; 2) Mandibular protrusion, defined as the displacement of the lower incisor from the position of Maximum Habitual Intercuspation (MHI) to maximum protrusion; and 3) Laterality, defined as the displacement of the lower incisor to the right and left from a central reference point aligned with the midline.

No post-hoc modifications to outcomes were made.

### Harms assessment

Adverse events were monitored systematically during all three assessment periods. Each participant was also directly questioned about any undesirable effects resulting from the use of phytocannabinoids.

### Randomisation

This study did not include random sequence generation. All participants underwent the same fixed sequence of interventions, receiving the placebo first, followed by the phytocannabinoid treatment. This decision was based on the study’s crossover, non-randomized pilot design and the exploratory nature of the investigation.

Although randomization is a core CONSORT element, its omission in crossover pharmacological pilot trials is acceptable when the sequence serves safety and mechanistic purposes, as in this study.

As the study followed a fixed crossover sequence (placebo followed by phytocannabinoid), no allocation concealment mechanism was applicable.

Participant recruitment and allocation to the fixed intervention sequence were conducted by the study coordinator, who was not involved in the clinical assessments. All clinical evaluations were performed by a blinded examiner.

### Blinding

The study was conducted under a double-blind design. Both participants and clinical examiners were blinded to the identity of the intervention. The placebo and phytocannabinoid extracts were identical in appearance, taste, and packaging, labeled only with coded identifiers prepared by an independent pharmacist. The code was kept sealed until completion of data analysis.

### Statistical methods

The data were tabulated and analyzed in the R environment (version 4.5). The statistical analysis of continuous outcomes (opening, protrusion, laterality, and pain intensity) was conducted using linear mixed models for repeated measures, applying the *lmer* function of the *lme4* package, and considering the correlation between the repeated measures of each participant.

The “Evaluation Time” factor (with three levels: T0, T1, T2) was included as a fixed effect, while the participants were modeled as a random effect, allowing the control of interindividual variability. Time points were compared using the *emmeans* package by calculating Estimated Marginal Means (EMMs), with Bonferroni adjustments applied for multiple comparisons. Significance was set at 5 % (*p* < 0.05).

The carryover or sequence effect was not specifically analyzed, as the design had a fixed order of intervention applications (placebo followed by cannabinoid) and no sequence randomizations.

## Results

### Participants, recruitment and follow-up

Twenty participants were assessed for eligibility, and all met the inclusion criteria. No participants were lost to follow-up or excluded after enrolment.

Recruitment occurred between 10/24 and 04/25. Each participant completed two consecutive 90-day intervention phases (placebo and phytocannabinoid).

### Baseline data

The study included 20 participants, of whom eight were men and twelve were women, aged between 29- and 61-years. All participants presented a clinical diagnosis of chronic myofascial pain based on the DC/TMD criteria.

### Outcomes and estimation

**Fig. 2:**
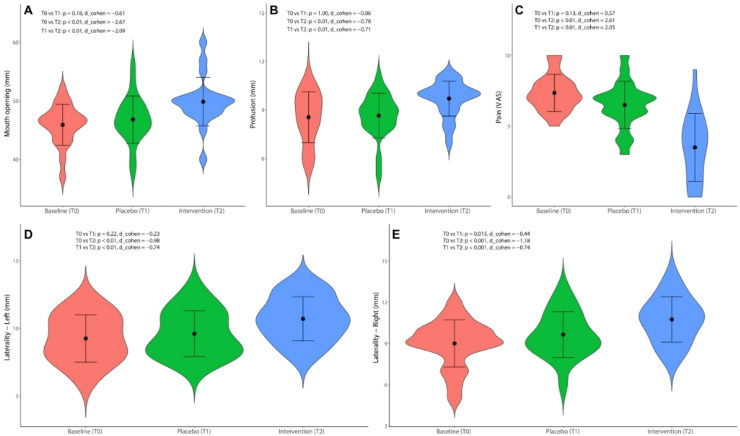
Violin plots show Baseline (T0), Placebo (T1), and Intervention (T2) results for five clinical outcomes: (A) Mouth opening; (B) Protrusion; (C) Pain (VAS), (D) Left laterality and (E) Right laterality. Each plot displays value distributions, means (black dot), and standard errors (black bars). Statistical comparisons, including p-values and Cohen’s *d*, are provided within each panel.

The cannabinoid therapy (T2) presented a statistically significant improvement in all evaluated outcomes compared to the baseline (T0) and the post-placebo period (T1) (*p* < 0.001) (Table 1), with a high magnitude effect (Cohen's *d* > 0.8). The placebo caused significant improvement only in the right laterality compared to the baseline (*p* = 0.01) (Fig. 2).

**Table 1:** Statistical analysis of the evaluated outcomes.

Outcome	Baseline (mean ± SD)	Placebo (mean ± SD)	Drug (mean ± SD)	p-value
Opening	45.9 ± 3.49ᵃ	46.8 ± 4.03ᵃ	49.9 ± 4.14ᵇ	<0.001
Protrusion	8.55 ± 1.57ᵃ	8.65 ± 1.45ᵃ	9.70 ± 1.46ᵇ	<0.001
Laterality ‒ R	9.00 ± 1.83ᵃ	9.65 ± 1.80ᵇ	10.75 ± 1.78ᶜ	<0.001
Laterality ‒ L	9.25 ± 1.78ᵃ	9.60 ± 1.80ᵃ	10.70 ± 1.80ᵇ	<0.001
VAS	7.35 ± 2.49ᵃ	6.50 ± 2.50ᵃ	3.50 ± 2.49ᵇ	<0.001

### Qualitative outcomes

The qualitative outcomes of allodynia, functional pain, hyperalgesia, and referred pain improved following cannabinoid therapy, with approximately a 90 % reduction in the number of patients reporting functional pain at T2 (Table 2). Given the small sample size, inferential statistical analyses were not conducted for these outcomes.

**Table 2:** Descriptive analysis of the outcomes of allodynia, functional pain, hyperalgesia and referred pain.

Time	Allodynia		Functional Pain		Hyperalgesia		Referred Pain	
	Yes	No	Yes	No	Yes	No	Yes	No
Baseline	6	14	20	0	13	7	20	0
Placebo	4	16	13	7	5	15	19	1
Drug	0	20	2	18	1	19	17	3

### Intervention fidelity and concomitant care

All participants adhered fully to the intervention protocol, verified through follow-ups every 20-days. No additional therapies for temporomandibular pain were used during the study.

No subgroup or sensitivity analyses were conducted.

### Adverse reactions

No adverse effects were reported, either in response to direct questioning or spontaneously.

## Discussion

Myofascial pain is a significant factor in TMDs, affecting a large part of the adult population and causing marked losses in quality of life.^13^ The most frequent management methods include Non-Steroidal Anti-inflammatory Drugs (NSAIDs), tricyclic antidepressants, neuroleptics, muscle relaxants, and physical interventions. These methods produce risks of long-term adverse effects from drugs and the discomfort associated with invasive techniques, reinforcing the need for additional therapies that work in an integrated manner on the peripheral, central, and psychosocial components of myofascial pain.^14^^,^^15^ In this context, this study evaluated the effects of combined sublingual Δ9-THC and CBD (10 mg/day, 1:1 ratio) therapy on myofascial pain modulation compared to placebo, over 180-days of follow-up.

The findings demonstrated a robust analgesic effect on all evaluated outcomes after using Δ9-THC/CBD. Regarding pain intensity, the reductions in the VAS were higher than those in pilot trials with CBD used alone for musculoskeletal diseases^16^ and dry needling protocols,^17^ which did not present concomitant functional gains. Reviews of cannabinoids in chronic musculoskeletal pain also reported lower decreases in pain than those of the present study, although they highlighted the good tolerability profile of these substances.^5^ These findings suggest a superior analgesic capacity of the combined Δ9-THC/CBD regimen and indicate functional synergy between the two phytocannabinoids in modulating nociception, in line with preclinical studies demonstrating lower nociceptive excitability and muscle inflammation by this combination.^18^

Although few clinical studies have directly quantified improvements in mandibular protrusion and lateral excursions using conventional or cannabinoid treatments, the findings of the present study highlight the relevance of cannabinoid therapy and verified functional gains. In this scenario, Vieira et al.^19^ evaluated the combined effect of manual therapy and physical exercise. Although the authors considered an outcome aimed at mouth opening, their findings were comparable to the gains observed in the present study for protrusion and lateral excursions (greater than 4 mm), suggesting that the combined Δ9-THC/CBD regimen may offer functional efficacy equivalent to that of conventional rehabilitation protocols. Similarly, a recent study on multiple sclerosis found that cannabinoids may significantly reduce muscle spasticity and help rehabilitate patients with debilitating symptoms of disability.^20^ Additionally, a systematic review by Walczyńska-Dragon et al.^21^ on the therapeutic potential of CBD in TMD and orofacial pain suggested that CBD may relieve TMD-related pain and muscle tension with minimal adverse effects. Although specific effects on protrusion and laterality have not been directly evaluated, reducing muscle pain and tension may help improve mandibular function.

The placebo effect in TMD treatment revealed partial improvement in right laterality, suggesting that non-treatment-specific factors are also relevant in perceiving symptom relief. Physiologically, the placebo effect can be explained by several neurophysiological mechanisms, such as opioid, endocannabinoid, oxytocin, vasopressin, and dopamine systems, which release endorphins and modulate pain pathways in the central nervous system. These mechanisms may be activated simply by patients believing they are receiving an effective treatment.^22^, ^23^, ^24^ Psychologically, patients' expectations regarding treatment effectiveness may trigger a pain relief response, which is amplified by the positive emotional aspect associated with confidence in the therapeutic process.^22^, ^23^, ^25^ These effects highlight the importance of strict blinding in clinical trials, as they ensure that patients are not influenced by their expectations of treatment. The placebo effect may be induced by verbal cues, classical and non-classical conditioning, and social interactions, which help form expectations based on previous experiences and the observation of others.^22^^,^^23^ Moreover, communication with participants regarding the nature of the study should be carefully managed to prevent an expectation bias, ensuring the validity of results and minimizing the impact of the placebo effect on the evaluated outcomes.^26^

The analysis of qualitative outcomes of allodynia, hyperalgesia, and functional pain presented a substantial decrease in the prevalence of these symptoms among participants undergoing cannabinoid therapy. The relevant reduction in the number of patients who reported functional pain after treatment with sublingual Δ9-THC and CBD may suggest that the therapy impacts pain relief associated with myofascial pain and other chronic musculoskeletal disorders. This effect is particularly significant considering the current scenario of conventional TMD treatments, which often have limitations, especially concerning adverse effects.^3^

Cannabinoids, particularly Δ9-tetrahydrocannabinol (Δ9-THC) and Cannabidiol (CBD), have shown significant potential in modulating myofascial pain by acting on multiple mechanisms in the peripheral and central nervous systems.^27^ Δ9-THC, an agonist of cannabinoid receptors CB1 and CB2, provides an analgesic effect by modulating pain transmission, reducing nociceptive activity in the central nervous system, and decreasing peripheral inflammation.^28^^,^^29^ CBD, in turn, acts as an indirect modulator of these receptors, promoting anti-inflammatory and analgesic effects with a more pronounced influence on CB2 receptors in peripheral tissues, such as skeletal muscles, suggesting a direct therapeutic effect on myofascial pain.^21^ Together, these compounds demonstrate a synergistic effect, reducing inflammation and pain with a complementary action in controlling systemic and local inflammatory responses.^30^ The activation of CB1 receptors in the central nervous system may interfere with pain perception, and the stimulation of peripheral CB2 receptors has effectively modulated inflammatory signals, which is crucial in chronic musculoskeletal pain.^31^ These mechanisms are particularly relevant in TMD, where myofascial pain is a common condition associated with changes in masticatory muscles and local inflammation. Therefore, cannabinoids have a high therapeutic potential in managing these conditions by offering an alternative for patients with chronic musculoskeletal pain who do not respond adequately to conventional treatments. These substances may also improve factors such as stress and anxiety, often present in the maintenance of chronic pain.^32^

This study has limitations inherent to its non-randomized design and the absence of a washout period between phases, which could potentially lead to biases related to sequence and carryover effects. However, the likelihood of these effects occurring is minimal, as only the patient was blinded, and the study began with the use of a placebo. This design allowed the evaluator to be confident that there was no carryover, without compromising the internal validity of the findings. The methodological decision to use a fixed order was based on ethical and operational restrictions. The temporal extension of the phases and the continuous longitudinal monitoring of participants sought to minimize residual interference, although without a specific statistical analysis for the carryover effect. The duration of each phase (90-days for the placebo and 90-days for the pharmacological intervention) was also planned to reduce overlapping effects. Additionally, the absence of a valid inferential statistical analysis for allodynia, hyperalgesia, and functional pain indicates that, although the findings are promising, caution is needed when interpreting them. The small number of reported events hindered more detailed comparisons and the generalization of results to broader populations. This limitation is frequent in pilot studies and underscores the need for larger sample sizes and greater statistical power in future research.^10^ Another limitation of this study is the absence of stress and anxiety assessments. While participants’ experiences were qualitatively explored, quantifying the emotional impact of the intervention and examining its associations with clinical outcomes would provide a more comprehensive understanding of the potential effects of phytocannabinoids. Future studies should be adequately powered and incorporate these outcomes to strengthen the evaluation of emotional state changes associated with this therapy.

Conversely, the employed crossover design enabled the control of interindividual variability, increasing the estimated accuracy of intervention effects using a relatively small sample. Validated and standardized clinical instruments for measuring pain intensity, myofascial sensitivity, and mandibular functional parameters, associated with rigorous prospective follow-up, provide methodological robustness and reliability to the outcomes.

Although the preliminary findings, such as those in this study, suggest an improvement in functional pain, further studies on the effects of cannabinoids on TMD and other musculoskeletal conditions are necessary for a better understanding of the efficacy, action mechanisms, and adverse effects of this therapy. Regarding future investigations, crossover randomized clinical trials with sequence randomization and inclusion of appropriate washout periods are recommended to mitigate biases and improve internal validity. Expanding the sample size and incorporating clinical biomarkers may broaden the generalization and understanding of the action mechanisms of cannabinoids in the context of TMD.

## Conclusion

Cannabinoid therapy was effective in reducing painful symptoms in TMD patients, associated with relevant functional improvements in mandibular opening, protrusion, and laterality compared to placebo. These findings indicate the clinical potential of cannabinoids as a promising therapeutic alternative for managing TMD, highlighting the need for future studies with larger samples and randomized designs to validate and enhance the action mechanisms.

## Ethical approval

The Human Research Ethics Committee of São Leopoldo Mandic College (SLMADIC) approved the research under opinion n° 6.317.731 and CAAE: 71,674,023.8.0000.5374, complying with the ethical principles of the Declaration of Helsinki and Brazilian national regulatory standards.

## Funding

This study was financed in part by the Coordenação de Aperfeiçoamento de Pessoal de Nível Superior – Brazil (CAPES) – Finance Code 001. Support from the Conselho Nacional de Desenvolvimento Científico e Tecnológico – Brazil (CNPq) is also gratefully acknowledged.

## Data availability statement

The datasets generated and analyzed during the current study are available within the article. Raw data that support the findings of this study are available from the corresponding author upon reasonable request.

## CRediT authorship contribution statement

**Francisco Gomes Bonetto Schinko:** Conceptualization, Methodology, Writing – original draft, Writing – review & editing, Project administration, Supervision. **Luiz Renato Paranhos:** Methodology, Investigation, Data curation, Formal analysis, Writing – original draft, Writing – review & editing. **Lucas Gonçalves de Sousa:** Methodology, Investigation, Data curation, Formal analysis, Writing – original draft, Writing – review & editing. **Gabriel Phelipe de Paula Santos:** Methodology, Investigation, Data curation, Formal analysis, Writing – original draft, Writing – review & editing. **Sigmar de Mello Rode:** Methodology, Investigation, Data curation, Formal analysis, Writing – original draft, Writing – review & editing. **Antonio Sergio Guimarães:** Conceptualization, Methodology, Writing – original draft, Writing – review & editing, Project administration, Supervision. **Juliana Cama Ramacciato:** Conceptualization, Methodology, Writing – original draft, Writing – review & editing, Project administration, Supervision.

## Declaration of competing interest

The authors declare no conflicts of interest.
